# Roles of TLR7 in Activation of NF-κB Signaling of Keratinocytes by Imiquimod

**DOI:** 10.1371/journal.pone.0077159

**Published:** 2013-10-11

**Authors:** Zheng Jun Li, Kyung-Cheol Sohn, Dae-Kyoung Choi, Ge Shi, Dongkyun Hong, Han-Eul Lee, Kyu Uang Whang, Young Ho Lee, Myung Im, Young Lee, Young-Joon Seo, Chang Deok Kim, Jeung-Hoon Lee

**Affiliations:** 1 Department of Dermatology and Research Institute for Medical Sciences, School of Medicine, Chungnam National University, Daejeon, Korea; 2 Department of Dermatology, The First Affiliated Hospital of Guangdong Medical College, Zhanjiang, China; 3 Department of Dermatology, Soonchunhyang University College of Medicine, Cheonan, Korea; 4 Department of Anatomy, School of Medicine, Chungnam National University, Daejeon, Korea; Yong Loo Lin School of Medicine, National University of Singapore, Singapore

## Abstract

Imiquimod is known to exert its effects through Toll-like receptor 7 (TLR7) and/or TLR8, resulting in expression of proinflammatory cytokines and chemokines. Keratinocytes have not been reported to constitutively express TLR7 and TLR8, and the action of imiquimod is thought to be mediated by the adenine receptor, not TLR7 or TLR8. In this study, we revealed the expression of TLR7 in keratinocytes after calcium-induced differentiation. After addition of calcium to cultured keratinocytes, the immunological responses induced by imiquimod, such as activation of NF-κB and induction of TNF-α and IL-8, were more rapid and stronger. In addition, imiquimod induced the expression TLR7, and acted synergistically with calcium to induce proinflammatory cytokines. We confirmed that the responses induced by imiquimod were significantly inhibited by microRNAs suppressing TLR7 expression. These results suggest that TLR7 expressed in keratinocytes play key roles in the activation of NF-κB signaling by imiquimod, and that their modulation in keratinocytes could provide therapeutic potential for many inflammatory skin diseases.

## Introduction

Recent studies have shown that engagement of Toll-like-receptors (TLRs), types of pattern recognition receptors that recognize pathogen-associated molecular patterns present in microbes, play significant roles in both innate and adaptive immunity [1]–[3]. In humans, at least 10 TLRs have been identified. TLRs can be divided into surface-expressed TLRs (TLRs 1, 2, 4, 5, 6, and 10), which are receptors for lipid-based ligands, and intracellular TLRs (TLRs 3, 7, 8, and 9), which are receptors for nucleic-acid-based ligands [4], [5]. TLR7 and TLR8, for instance, recognize nucleic acid types found in viruses. Moreover, the responsiveness of nucleic acid-sensing TLRs has a profound impact on immune responses, leading to the production of pro-inflammatory cytokines and up-regulation of co-stimulatory molecules necessary for the adaptive immune response [6]–[8].

In the skin, cells of the innate immune defense system recognize the pathogen-associated molecular structures via various TLRs [9], [10]. Keratinocytes also express various TLRs, and participate in innate immune response actively. Stimulation of keratinocytes with TLR ligands results in different immunological responses, leading to nuclear translocation of nuclear factor (NF)-κB and resulting in the production of interleukin (IL)-8, macrophage inflammatory protein-3a (CCL20), and cutaneous T-cell-attracting chemokine (CCL27) [11], [12].

Imiquimod (R837) is a specific TLR7 ligand. Imiquimod as a 5% cream is used to treat several skin diseases, including malignant melanoma, basal cell carcinoma, and squamous cell carcinoma [13]–[15]. Expression of TLRs 1, 2, 3, 4, 5, 6 and 9 (but not TLR7 and TLR8) has been reported in keratinocytes [16]–[18]. Nevertheless, imiquimod, a well-known ligand for TLR7 and TLR8, activates NF-κB and enhances the expression of IL-6, IL-8, tumor necrosis factor (TNF)-α, and IL-1β in keratinocytes. Thus, imiquimod is thought to activate keratinocytes by binding to adenoid receptors in keratinocytes, independent of TLR7 and TLR8. The importance of imiquimod in inflammation prompted us to investigate the roles of TLR7 in calcium-differentiated keratinocytes.

In this study, we examined the expression of TLR7 in keratinocytes, and the effect of imiquimod on NF-κB signaling via these receptors. Our data provide compelling evidence that imiquimod stimulates NF-κB, IL-8 and TNF-α expression in keratinocytes via TLR7, and it is tempting to propose that modulation of these pathways could be an interesting candidate for the treatment of inflammatory skin diseases.

## Results

**Figure 1:**
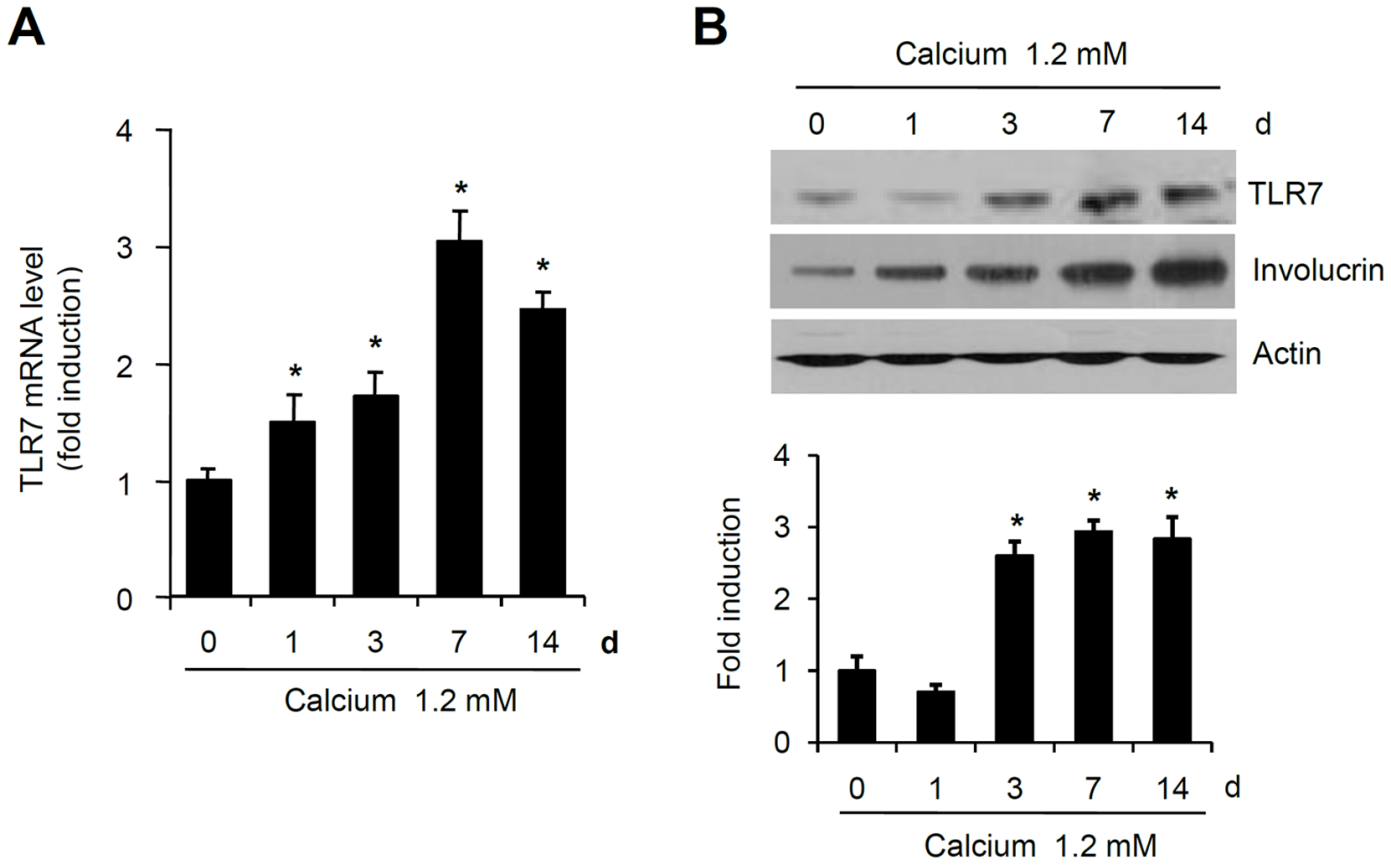
Expression of TLR7 in differentiated keratinocytes. (A) Keratinocytes were differentiated in the presence of 1.2 mM calcium. Cells were harvested at 1, 3, 7, and 14 days. TLR7 mRNA expression were assessed by real-time PCR analysis. Relative expression level was standardized using cyclophilin as an internal control. (B) Whole-cell extracts of keratinocytes that had undergone calcium-induced differentiation were subjected to Western blot analysis. Quantification of Western bands were done using Densitometer. Data are expressed as fold induction ± SE (n = 3). *P<0.05 versus non-calcium-treated cells.

In this study, we evaluated the expression of TLR7 during keratinocyte differentiation by adopting a well-established calcium-induced differentiation model [19]. Real-time PCR analysis clearly showed that the expression of TLR7 increased during the 7 days after calcium treatment (Figure 1A). Protein analysis by Western blotting further identified endogenous TLR7 expression in keratinocytes treated with calcium (Figure 1B).

**Figure 2:**
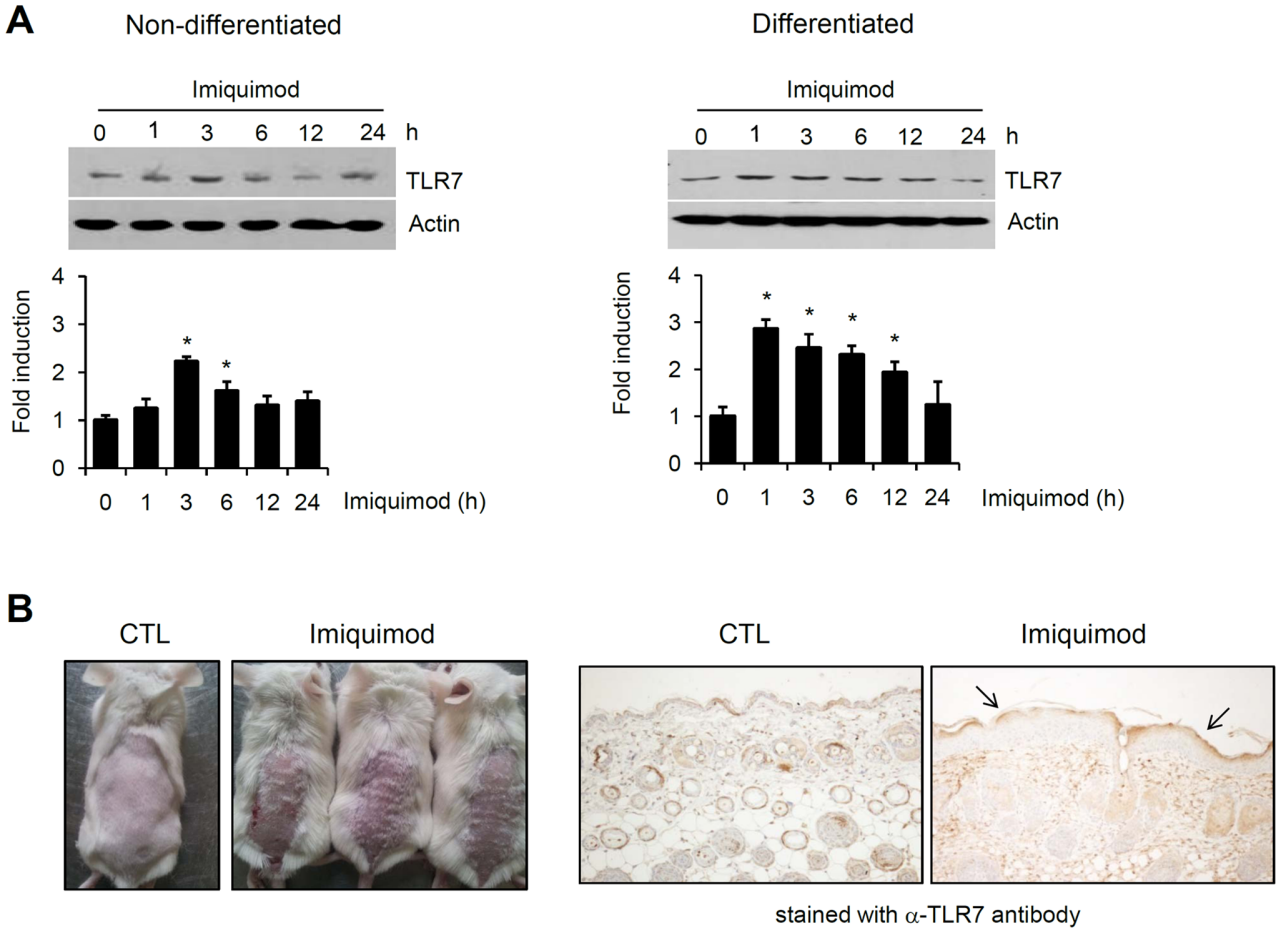
Imiquimod induces TLR7 expression in keratinocytes. (A) Keratinocytes were grown in low calcium condition (non-differentiated) or 1.2 mM calcium condition for 7 days (differentiated). Cells were treated with 100 µM imiquimod for the indicated time points, and were then assessed for TLR7 by Western blotting. Quantification of Western bands were done using Densitometer. Data are expressed as fold induction ± SE (n = 3). *P<0.05 versus non-calcium-treated cells. (B) Aldara (5% imiquimod cream) was applied to the back skin of 6 week old ICR mouse for 7 days. Paraffin section was stained with anti-TLR7 antibody. Arrows indicate the TLR7 expression is increased in upper epidermis.

It has been demonstrated that keratinocytes stimulated with imiquimod exhibit nuclear translocation of NF-κB and release of TNF-α and IL-8 [11]. We thought that imiquimod can induce the expression of TLR7 in keratinocytes. Therefore, we investigated the effects of calcium on the actions of imiquimod in keratinocytes. We found that imiquimod induced the expression of TLR7 in both non-differentiated keratinocytes and differentiated keratinocytes by calcium treatment for 7 days (Figure 2A, Figure S1). Consistent with this result, TLR7 expression was markedly increased in upper epidermal layer of imiquimod-treated mouse skin (Figure 2B).

**Figure 3:**
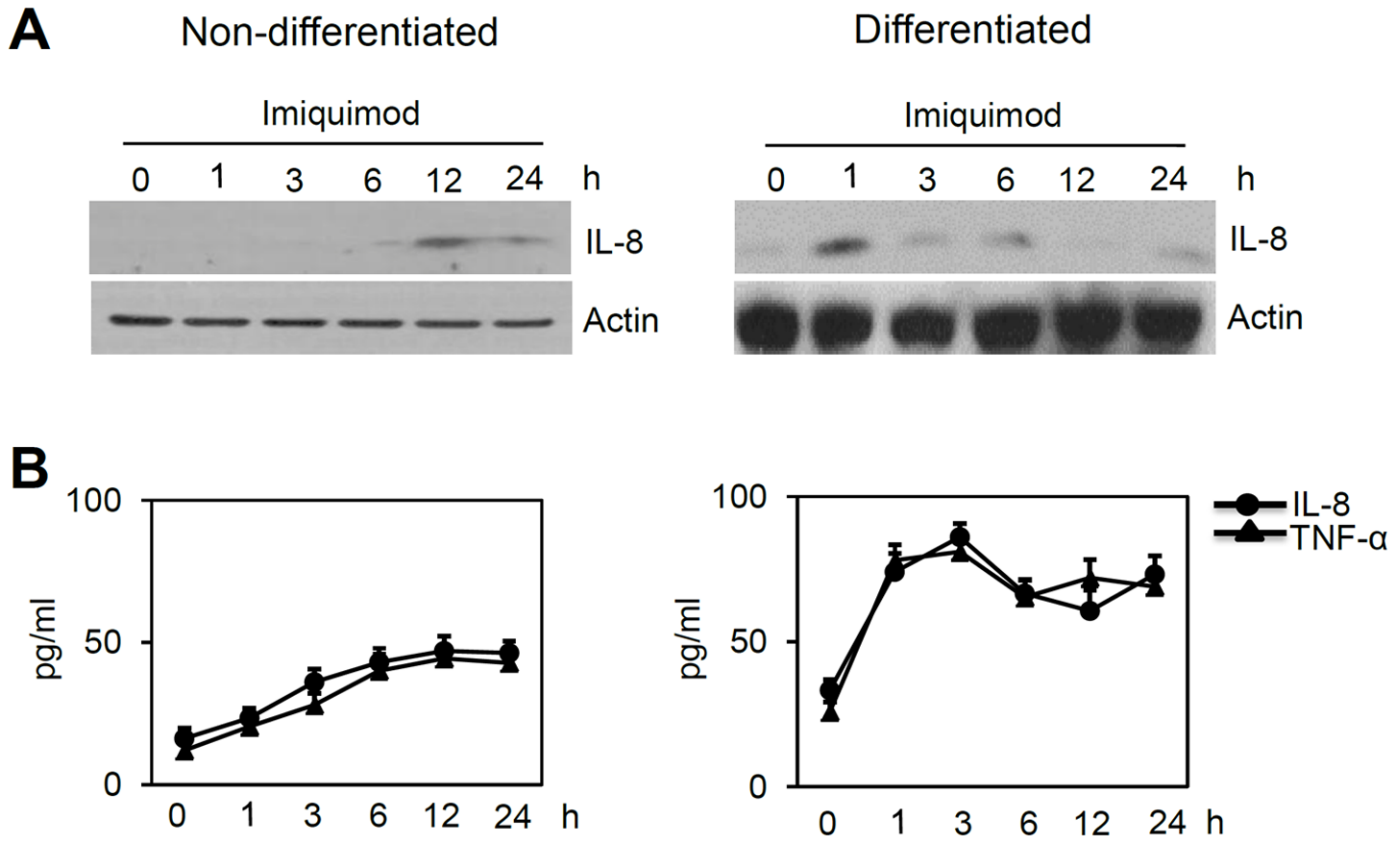
Imiquimod induces cytokine expression in keratinocytes. (A) Keratinocytes grown in low- and high-calcium (day 7) media were treated with 100 µM imiquimod for the indicated time points, and were then assessed for TLR7 by Western blotting. (B) IL-8 and TNF-α were evaluated by ELISA. Data represent the mean ± SE (n = 3).

When keratinocytes were grown in low-calcium medium (non-differentiated keratinocytes), imiquimod treatment resulted in induction of IL-8 and TNF-α at relatively late time points. Interestingly, in cells grown in high-calcium medium (differentiated keratinocytes), expression of IL-8 and TNF-α peaked at relatively early time points after stimulation of imiquimod, with higher amount compared to non-differentiated keratinocytes (Figure 3, Figure S2). In addition, expression of other cytokines including CCL20, interferon-α, and interferon-β was also increased by imiquimod in a similar way (Figure S3). These results were consistent with increased expression of TLR7 after calcium treatment in cultured keratinocytes. Thus, the effects of imiquimod on skin, which include antiviral activity and exacerbation of psoriasis, may be mediated through TLR7 in synergy with calcium.

**Figure 4:**
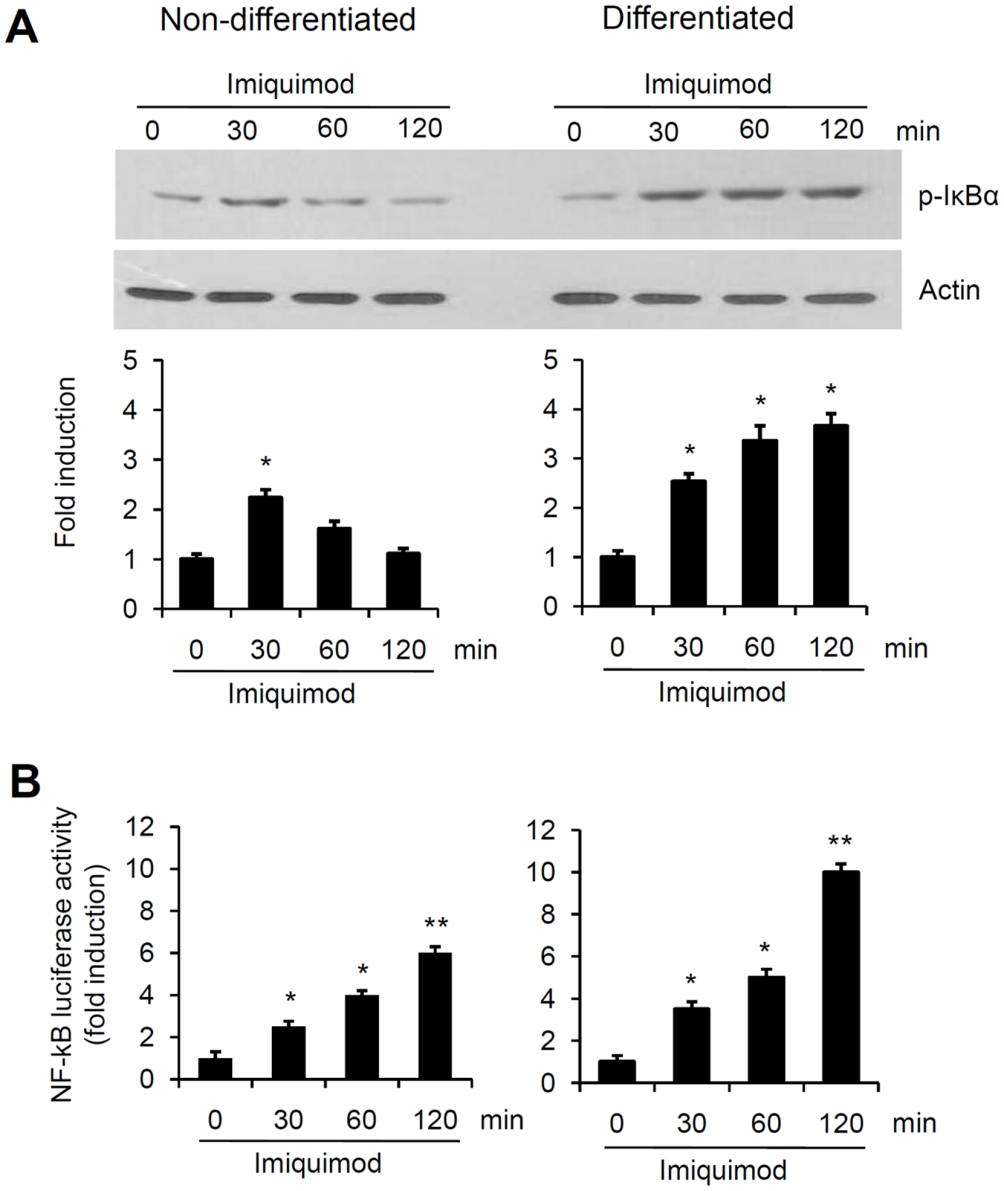
Imiquimod increases NF-κB transcriptional activity in keratinocytes. (A) Keratinocytes were stimulated with 100 µM imiquimod for the indicated time points. Cell lysates were assessed by Western blotting using an antibody against phospho-IκBα. Quantification of Western bands were done using Densitometer. Data are expressed as fold induction ± SE (n = 3). *P<0.05 versus non-calcium-treated cells. (B) Keratinocytes were transduced with NF-κB reporter adenovirus and then treated with 100 µM imiquimod. Luciferase assays were performed and data are expressed as fold induction. Data represent the mean ± SE (n = 3). *P<0.05, **P<0.01.

Next, we assessed whether calcium could affect triggering of a typical TLR-related signaling response by TLR7: the phosphorylation of IκBα in keratinocytes. IκBα phosphorylation peaked at 30 min and then declined gradually in keratinocytes grown in low calcium-medium (non-differentiated) after treatment with imiquimod. When keratinocytes were induced to differentiate by exposure to calcium for 7 days and then stimulated with 100 µM imiquimod, phosphorylation of IκBα prolonged up to 120 min (Figure 4A). To check whether this IκBα phosphorylation resulted in NF-κB transcriptional activation, cells were transduced with an NF-κB reporter adenovirus and then stimulated with 100 µM imiquimod. Imiquimod led to the more rapid and persistent activation of NF-κB when cells were treated with calcium (Figure 4B).

**Figure 5:**
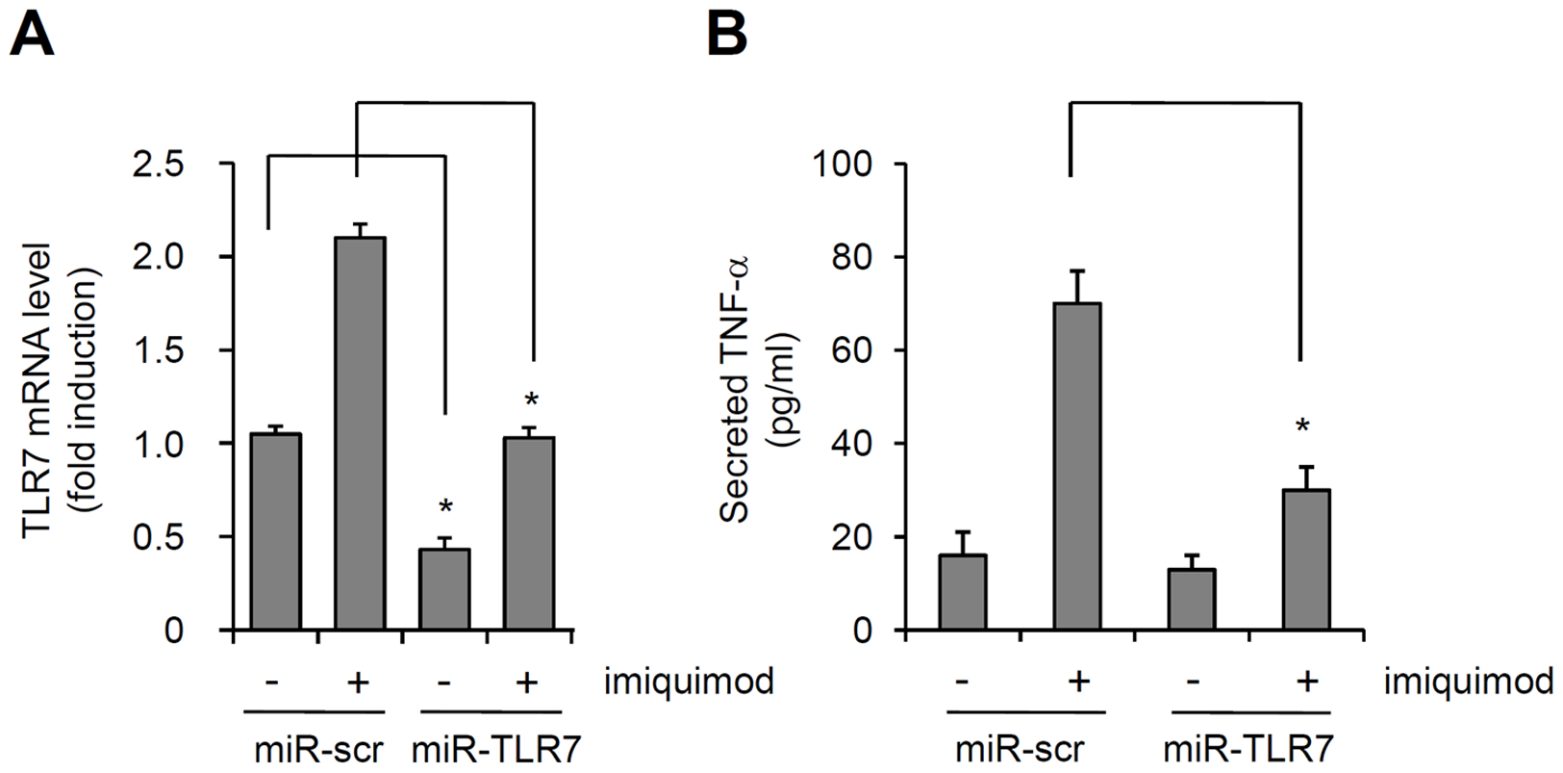
Imiquimod induces the expression of TNF-α via TLR7. Keratinocytes cultured in 1.2(day 7) were transduced with adenoviruses expressing miR-scr and miR-TLR7, and then treated with 100 µM imiquimod. (A) TLR7 mRNA expression were assessed by real-time PCR analysis. Relative expression level was standardized using cyclophilin as an internal control. (B) TNF-α secretion was assessed by ELISA. Data represent the mean ± SE (n = 3). *P<0.05.

We determined the effect of TLR7 knockdown on imiquimod-induced cytokine expression. Keratinocytes were cultured in the presence of calcium for 7 days, transduced with adenoviruses expressing miR-scr and/or miR-TLR7, and then treated with imiquimod. The microRNA suppressed TLR7 in keratinocytes that underwent calcium-induced differentiation (Figure 5A). As expected, knockdown of TLR7 significantly inhibited the imiquimod-induced TNF-α production (Figure 5B).

**Figure 6:**
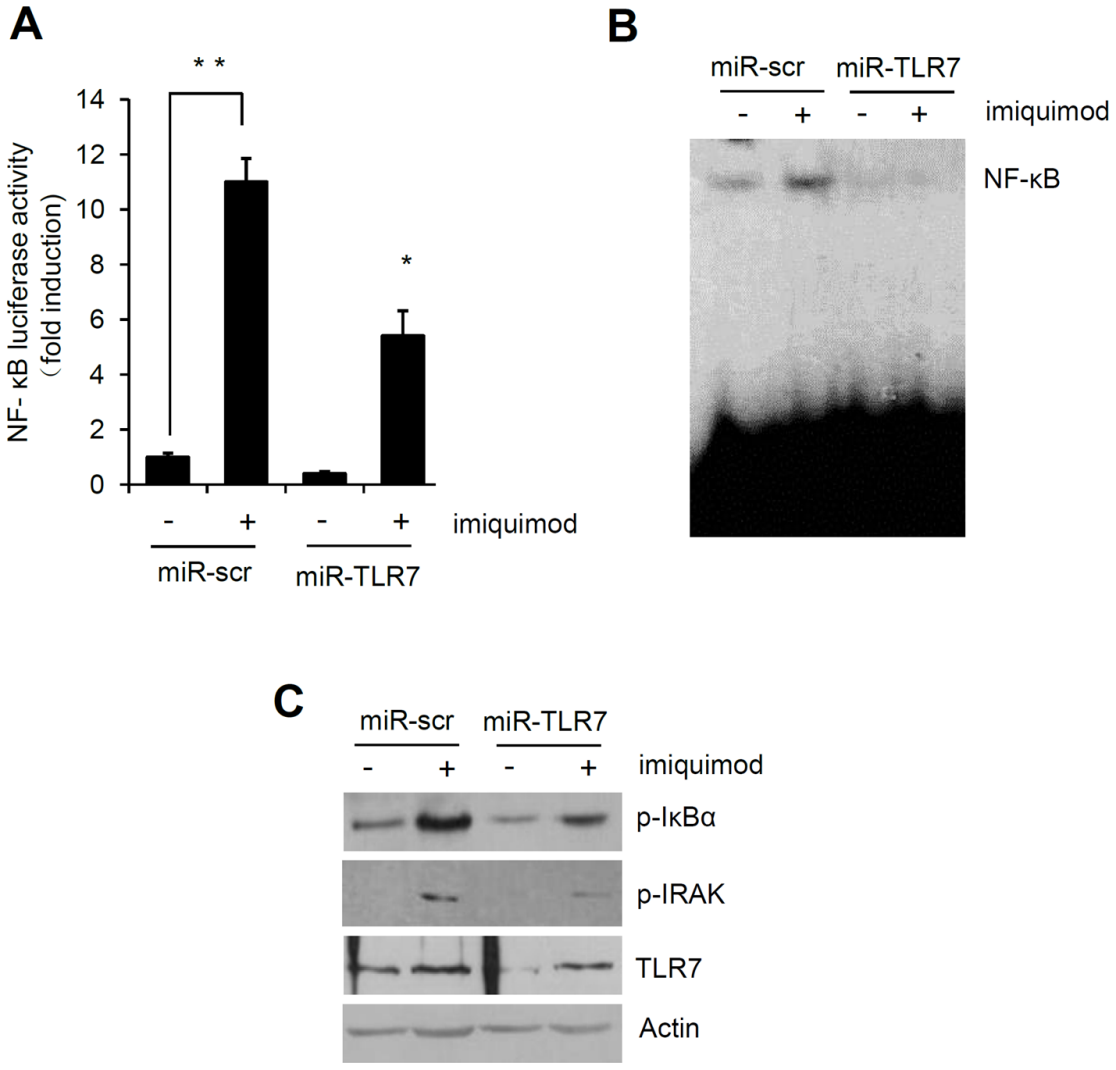
Imiquimod induces activation of NF-κB via TLR7. (A) Keratinocytes cultured in 1.2 mM calcium (day 7) were transduced with adenoviruses expressing miR-scr and miR-TLR7, together with NF-κB reporter adenovirus. After treatment with 100 µM imiquimod, luciferase assays were performed. Data are expressed as fold induction. Data represent the mean ± SE (n = 3). *P<0.05, **P<0.01. (B) Nuclear extracts were used for evaluation of NF-κB binding activity by EMSA. The specific bands indicate the binding of NF-κB in nuclear extracts from cells treated with 100 µM imiquimod. (C) Cells were transduced with adenoviruses expressing miR-scr and miR-TLR7, and then treated with 100 µM imiquimod. Protein samples were assessed in terms of p-IκBα and p-IRAK expression by Western blotting.

We examined whether inhibition of TNF-α expression was attributable to decreased NF-κB transcriptional activity. After inducing the differentiation by calcium for 7 days, keratinocytes were co-transduced with NF-κB luciferase reporter adenovirus and microRNA expressing adenovirus, and then treated with imiquimod. Knockdown of TLR7 significantly reduced NF-κB transcriptional activity (Figure 6A). The NF-κB binding activity was confirmed by electrophoretic mobility shift assay (EMSA), the results of which showed that imiquimod-induced NF-κB binding activity was dramatically reduced by miR-TLR7. Even endogenous transcriptional activity of NF-κB was also significantly inhibited by miR-TLR7 (Figure 6B). We also showed that imiquimod increased phosphorylation of IκBα and IRAK, a response that was inhibited by miR-TLR7 (Figure 6C). In addition, imiquimod increased some NF-κB downstream molecules such as CIAP2 and XIAP, and TLR7 knockdown significantly blocked the imiquimod-induced CIAP2 and XIAP expression (Figure S4). Taken together, our data indicate that imiquimod activates NF-κB in TLR7-dependent manner.

## Discussion

Imiquimod has been demonstrated to exert its biological activity through direct activation of TLR7 and/or TLR8, both of which were recently identified as natural receptors for single-stranded RNA [11], [20], [21]. Previously, no expression of TLR7 or TLR8 in human keratinocytes, the major cells in the epidermis of the skin, was reported. It has been proposed that the anti-viral and anti-tumor effects of imiquimod in the skin are mediated by activation of TLR7 and/or TLR8 expressed by monocytes, macrophages, and plasmacytoid dendritic cells (pDCs). However, stimulation of keratinocytes by imiquimod also results in increased cytokine production [22], [23]. TLR-independent effects of imiquimod have been suggested to stem from its interference with adenosine receptor signaling mediated by adenylyl cyclase. Nevertheless, imiquimod was also shown to exert direct or indirect adenosine receptor-independent inhibition of adenylyl cyclase activity [11]. In contrast to previous study, our results suggest that the innate receptors TLR7 are expressed in cultured keratinocytes, particularly in differentiated keratinocytes. Moreover, expression of TLR7 could be induced in keratinocytes by imiquimod itself, potentiating its inflammatory response. Although the roles of TLR7 in keratinocytes in vivo remain unclear, our results suggest that keratinocytes play critical roles in the pathogenesis of several skin diseases, such as psoriasis, through TLR7.

The epidermis is a rich source of genomic DNA and RNA that can be released by trauma, infection, and normal differentiation [24]. Recently, activation of human dendritic cells through TLR7 and TLR8 by self RNA-antimicrobial peptide complexes provided new insights into the mechanism that provokes the auto-inflammatory responses in psoriasis [25]. Because imiquimod is an analogue of adenosine, it is interesting that it has been described to induce psoriasiform dermatitis in humans and mice when applied topically [6]. Psoriasiform dermatitis was originally attributed to upregulation of the type I interferon pathway by imiquimod, accompanied by the infiltration of plasmacytoid dendritic cells [6], [26], [27]. More recently, imiquimod-induced dermatitis was reported to be critically dependent on the IL-17 and IL-23 axis, which is known to play a crucial role in psoriasis [28]. Previously, stimulation of keratinocytes by imiquimod was thought to be TLR-independent. However, our results indicate that TLR7 in keratinocytes could play an important role in the pathogenesis of skin inflammatory diseases. In view of our findings, we suggest that the ability of imiquimod to activate keratinocytes via TLR7 could lead to psoriasis, supporting the notion that keratinocyte products influence immune activation [29], [30]. Our findings uncover a previously underappreciated role for keratinocytes in modulating the immune response in psoriasis. Regulation of TLR7 in keratinocytes could be potential therapeutic strategies for treating skin inflammatory diseases.

In conclusion, our study discovered expression of TLR7 in keratinocytes during calcium-induced differentiation, and that their ligand imiquimod induced activation of NF-κB, and thereby induced the expression of multiple cytokines. Our data strongly suggest that keratinocytes are important in the defense against invading pathogens on the skin and contribute to induction of the immune response through activation of TLR7, resulting in differential patterns of expression of genes involved in inflammatory responses. It further suggests that TLR7 and its ligand may contribute to therapy of some skin diseases.

## Materials and Methods

### Ethics Statement

All human skin samples were obtained under the written informed consent of donors, in accordance with the ethical committee approval process of the Institutional Review Board of Chungnam National University School of Medicine. All animal tests were approved by the Institutional Review Board of Chungnam National University School of Medicine.

### Cell Culture

Primary epidermal keratinocytes were cultured according to the method previously reported [19]. Keratinocytes were maintained in keratinocyte-serum free medium (K-SFM) supplemented with epidermal growth factor (EGF) and bovine pituitary extract (Gibco BRL, Rockville, MD). To induce differentiation, 1.2 mM calcium were added and further incubated for the indicated time points.

### Reverse Transcription-polymerase Chain Reaction (RT-PCR)

Aliquots of a reverse transcription mixture were analyzed by PCR using specific primer sets. The following primers sequences were used: TLR7, forward 5′-CCACAACCAACTGACCACTG-3′ and reverse 5′-GATCACACT TTGGCCCTTGT-3′; IL-8, forward 5′-CCTTTCCACCCCAAATTTATCA-3′ and reverse 5′-TTTCTGTGTTGGCGCAGTGT-3′; TNF-α, forward 5′-TGCTCCTCACCCACACCAT-3′ and reverse 5′-GGAGGTTGACCTTGGTCTGGTA-3′; CCL20, forward 5′-CCACCTCTGCGGCGAAT-3′ and reverse 5′-AGAATACGGTCTGTGTATCCAAGACA-3′; interferon-α, forward 5′-GGCCTTGACCTTTGCTTTACTG-3′ and reverse 5′-CACAGAGCAGCTTGACTTGCA-3′; interferon-β, forward 5′-AACTTTGACATCCCTGAGGAGATT -3′ and reverse 5′-GCGGCGTCCTCCTTCTG-3′; cyclophilin, forward 5′-CTCCTTTGAGCTGTTTGCAG-3′ and reverse 5′-CACCACATGCTTGCCATCCA-3′. For quantitative real-time PCR, SYBR Green real-time PCR master mix (Invitrogen, Carlsbad, CA) was used according to the manufacture’s protocol.

### Determination of Cytokine and Chemokine Production by ELISA

To determine the cytokine release, supernatants were collected and frozen until used for ELISA assays. For detection of IL-8 and TNF-α, ELISA kits from BioSource Int. (Solingen, Germany) were used (sensitivity <2 pg and <0.2 pg per ml, respectively). ELISAs were performed according to the manufacturer’s instructions. All data were calculated from triplicate wells and are presented as a mean ± SD.

### Western Blotting

Cells were lysed in Proprep solution (Intron). Total protein was measured using a Bradford protein assay kit (Bio-Rad Laboratories, Hercules, CA). Samples were run on SDS-polyacrylamide gels, transferred onto nitrocellulose membranes and incubated with appropriate antibodies. Blots were then incubated with peroxidase-conjugated secondary antibodies, visualized by enhanced chemiluminescence (Intron). The following primary antibodies were used in this study: TLR7 (Enzo Life Science, Farmingdale, NY), IL-8, involucrin (Santa Cruz Biotechnologies, Santa Cruz, CA, USA), phospho-IκBα, phospho-IRAK (Cell Signaling Technology, Beverly, MA), actin (Sigma, St. Louis, MO, USA).

### Adenovirus Creation

For microRNA (miR) specific for TLR7, target sequences were designed using Invitrogen’s RNAi Designer. The double-stranded DNA oligonucleotides were synthesized and cloned into the parental vector pcDNA6.2-GW/EmGFP-miR (Invitrogen, Carlsbad, CA). In this vector system, miR sequence for target gene is located at the downstream of EmGFP coding sequence, allowing the identification of miR expressing cells by observing GFP under fluorescent microscope. The expression cassette for miR was moved into pENT/CMV vector, and then adenovirus was made as previously reported [31]. The miR sequences are as follows: miR-TLR7, top strand TGCTGTGAAATCGATCTCTACCAGATGTTTTGGCCACTGACTGACATCTGGTAGATCGATTTCA, bottom strand CCTGTGAAATCGATCTACCAGATGTCAGTCAGTGGCCAAAACATCTGGTAGAGATCGATTTCAC.

### Immunocytochemistry

Cells were fixed in 1% paraformaldehyde and permeabilized by treatment with 0.25% TritonX-100. Cells were then incubated with 10 µg/ml of the anti-TNF-α antibody (Santa Cruz Biotechnology) at 4°C for overnight, washed twice in PBS, and incubated with R-phycoerythrin-conjugated secondary antibody (Santa Cruz Biotechnology). Images were obtained using an Olympus BX41 fluorescent microscope (Scientific Instrument Company, Temecula, CA).

### Luciferase Reporter Assay

Keratinocyte cells were seeded in 6-well dishes, then transduced with NF-κB reporter adenovirus together with micro RNAs expressing adenoviruses. In some experimental group, cells were also treated with 100 µM imiquimod. Cells were harvested and luciferase activity was measured using the dual luciferase reporter assay system (Promega, Madison, WI).

### Electrophoretic Mobility Shift Assay

Ten µg of nuclear extract was incubated with ^32^P-end-labeled, double-stranded NF-κB oligonucleotide (5′-AGTTGAGGGGACTTTCCCAGGC-3′) (Promega, Madison, WI) for 20 min at room temperature. Subsequently, the DNA–protein complexes were resolved in a 6.6% native polyacrylamide gel, and visualized by autoradiography.

### Statistical Analysis

All experiments were repeated at least three times with different batches of cells. Data were evaluated statistically using Student’s t-test. Statistical significance was set at P<0.05.

## Supporting Information

Figure S1 Keratinocytes were grown in low calcium condition (non-differentiated) or 1.2 mM calcium condition for 7 days (differentiated). Cells were treated with 100 µM imiquimod for the indicated time points. TLR7 mRNA expression were assessed by real-time PCR analysis. Relative expression level was standardized using cyclophilin as an internal control. Data are expressed as fold induction ± SE (n = 3).
(PDF)

Figure S2 Keratinocytes were grown in low calcium condition (non-differentiated) or 1.2 mM calcium condition for 7 days (differentiated). Cells were treated with 100 µM imiquimod for the indicated time points. (A) IL-8 mRNA and (B) TNF-α mRNA expressions were assessed by real-time PCR analysis. Relative expression level was standardized using cyclophilin as an internal control. Data are expressed as fold induction ± SE (n = 3).
(PDF)

Figure S3 Keratinocytes were grown in low calcium condition (non-differentiated) or 1.2 mM calcium condition for 7 days (differentiated). Cells were treated with 100 µM imiquimod for the indicated time points. (A) CCL20 mRNA, (B) interferon-α mRNA, and (C) interferon-β mRNA expressions were assessed by real-time PCR analysis. Relative expression level was standardized using cyclophilin as an internal control. Data are expressed as fold induction ± SE (n = 3).
(PDF)

Figure S4 Keratinocytes cultured in 1.2 mM calcium (day 7) were transduced with adenoviruses expressing miR-scr and miR-TLR7, and then treated with 100 µM imiquimod. (A) GFP expression is detected in adenovirus-transduced keratinocytes. miR expression is linked to GFP expression in adenovirus-transduced keratinocytes. (B) Western blot analysis for NF-κB downstream molecules, CIAP2 and XIAP. TLR7 knockdown significantly blocked the imiquimod-induced expression of CIAP1 and XIAP.
(PDF)
