# Supplementary figures and images for: Roles of TLR7 in Activation of NF-κB Signaling of Keratinocytes by Imiquimod
Source: PLoS One. 2013 Oct 11;8(10):e77159. doi: 10.1371/journal.pone.0077159 (PMC3795621; doi:10.1371/journal.pone.0077159)

Figure S1

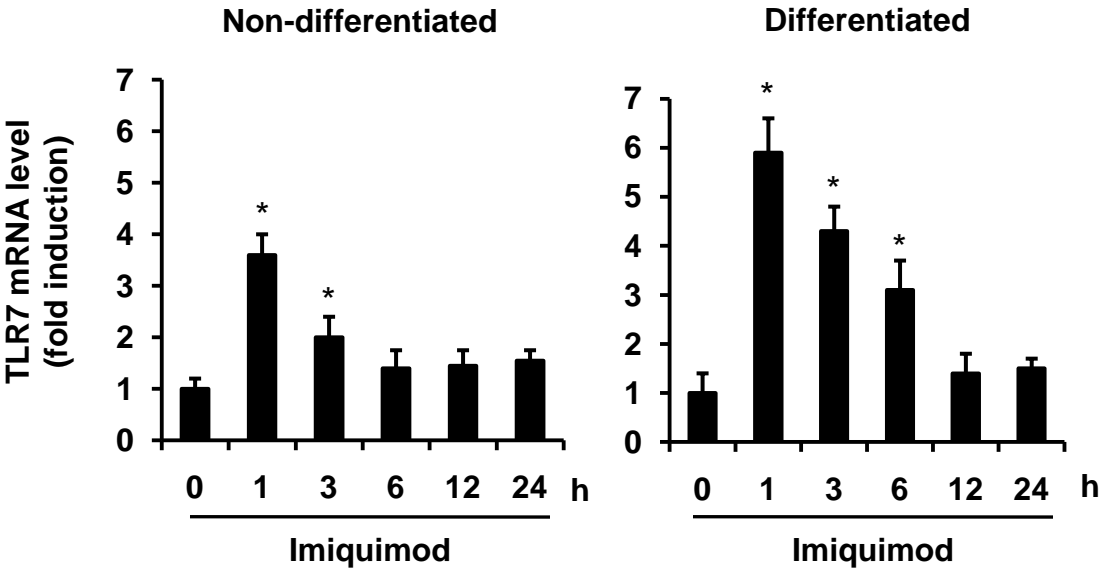

Supplement: Figure S1 — Keratinocytes were grown in low calcium condition (non-differentiated) or 1.2 mM calcium condition for 7 days (differentiated). Cells were treated with 100 µM imiquimod for the indicated time points. TLR7 mRNA expression were assessed by real-time PCR analysis. Relative expression level was standardized using cyclophilin as an internal control. Data are expressed as fold induction ± SE (n = 3). (PDF) [file pone.0077159.s001.pdf]

Figure S2

**A**

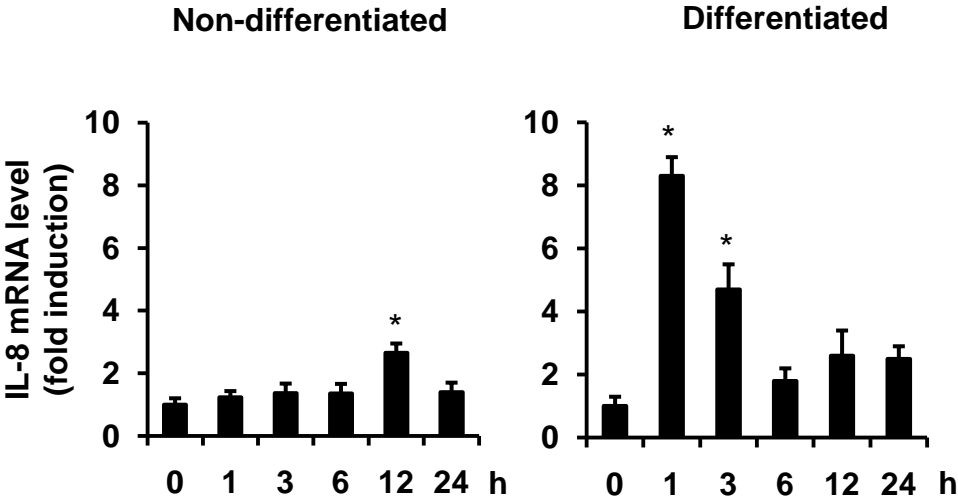

**B**

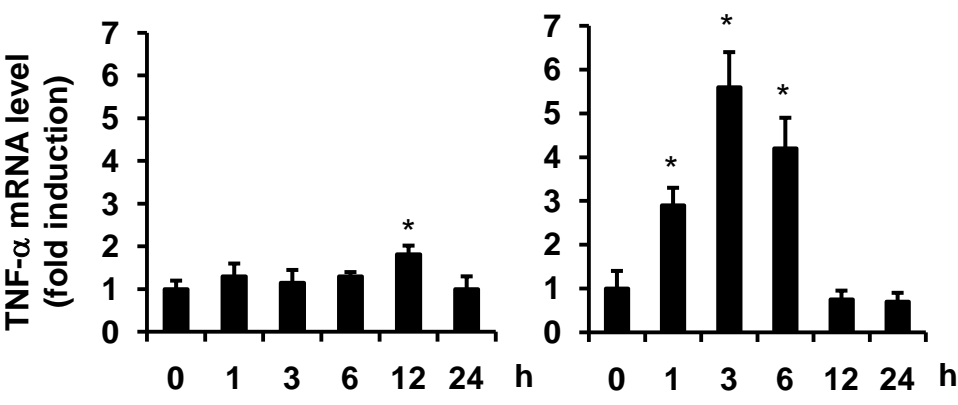

Supplement: Figure S2 — Keratinocytes were grown in low calcium condition (non-differentiated) or 1.2 mM calcium condition for 7 days (differentiated). Cells were treated with 100 µM imiquimod for the indicated time points. (A) IL-8 mRNA and (B) TNF-α mRNA expressions were assessed by real-time PCR analysis. Relative expression level was standardized using cyclophilin as an internal control. Data are expressed as fold induction ± SE (n = 3). (PDF) [file pone.0077159.s002.pdf]

Figure S3

**A**

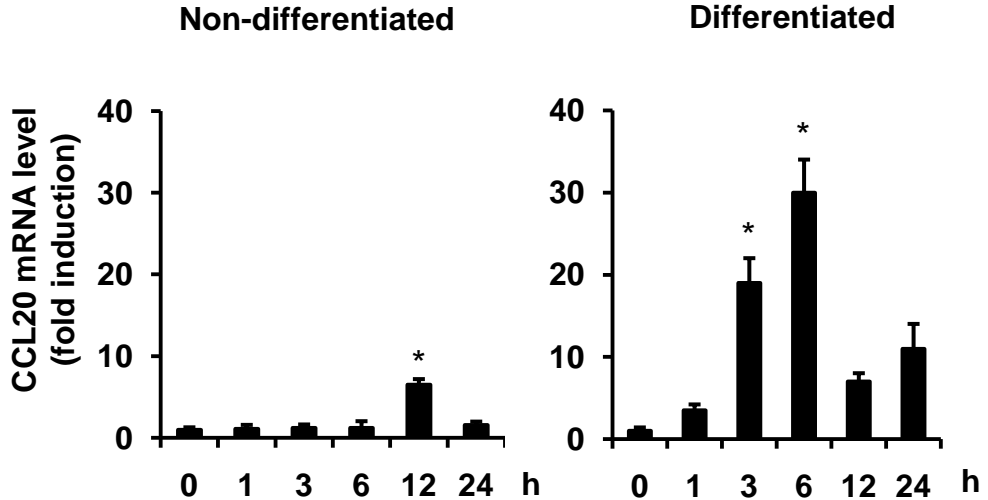

**B**

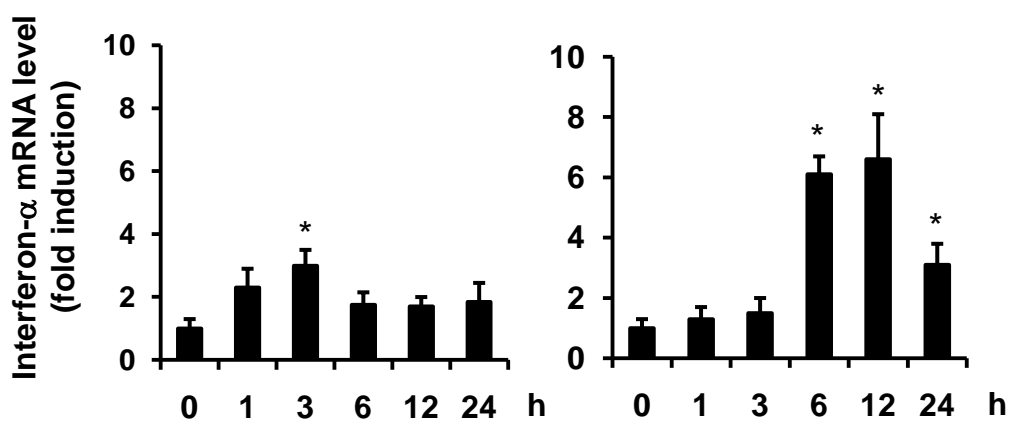

**C**

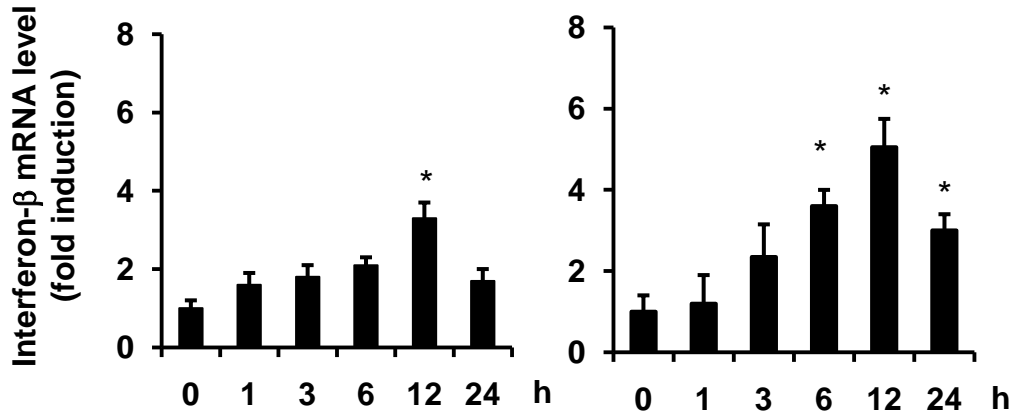

Supplement: Figure S3 — Keratinocytes were grown in low calcium condition (non-differentiated) or 1.2 mM calcium condition for 7 days (differentiated). Cells were treated with 100 µM imiquimod for the indicated time points. (A) CCL20 mRNA, (B) interferon-α mRNA, and (C) interferon-β mRNA expressions were assessed by real-time PCR analysis. Relative expression level was standardized using cyclophilin as an internal control. Data are expressed as fold induction ± SE (n = 3). (PDF) [file pone.0077159.s003.pdf]

Figure S4

**A**

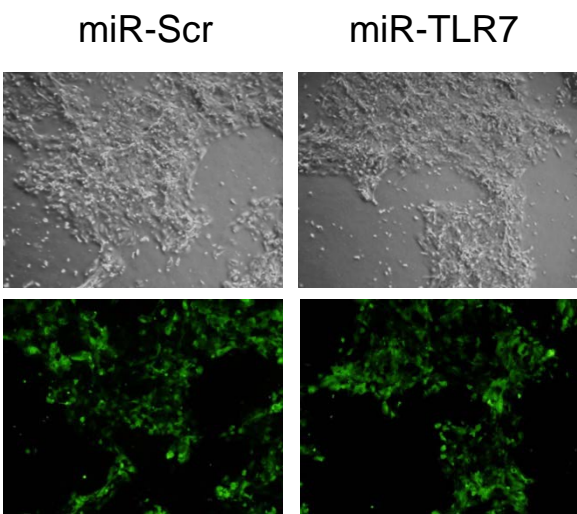

**B**

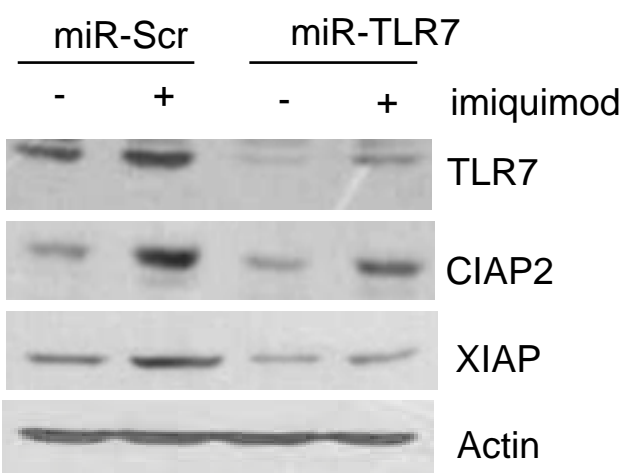

Supplement: Figure S4 — Keratinocytes cultured in 1.2 mM calcium (day 7) were transduced with adenoviruses expressing miR-scr and miR-TLR7, and then treated with 100 µM imiquimod. (A) GFP expression is detected in adenovirus-transduced keratinocytes. miR expression is linked to GFP expression in adenovirus-transduced keratinocytes. (B) Western blot analysis for NF-κB downstream molecules, CIAP2 and XIAP. TLR7 knockdown significantly blocked the imiquimod-induced expression of CIAP1 and XIAP. (PDF) [file pone.0077159.s004.pdf]
